# *Osteopathic Medicine and Primary Care *completes first year of publication

**DOI:** 10.1186/1750-4732-2-1

**Published:** 2008-01-24

**Authors:** John C Licciardone, Roberto Cardarelli

**Affiliations:** 1 Osteopathic Research Center, University of North Texas Health Science Center, Fort Worth, TX, USA; 2 Primary Care Research Institute, University of North Texas Health Science Center, Fort Worth, TX, USA

## Abstract

*Osteopathic Medicine and Primary Care *affords authors the opportunity for rapid and universal dissemination of their work. We are keen to receive author manuscripts and reader comments on articles during 2008. A journal fund has been established to offset the cost of article processing charges for eligible authors who submit qualified manuscripts.

## *Osteopathic Medicine and Primary Care*

In January 2007, we launched *Osteopathic Medicine and Primary Care *to provide rapid and universal dissemination of peer-reviewed research and scholarly work relevant to the full spectrum of primary care issues faced by a variety of clinicians and health care researchers, particularly those involving uniquely osteopathic aspects [1]. As we complete our first year of publication we are pleased to report on our progress and are excited about prospects for the coming year.

## Open access publishing

**Figure 1:**
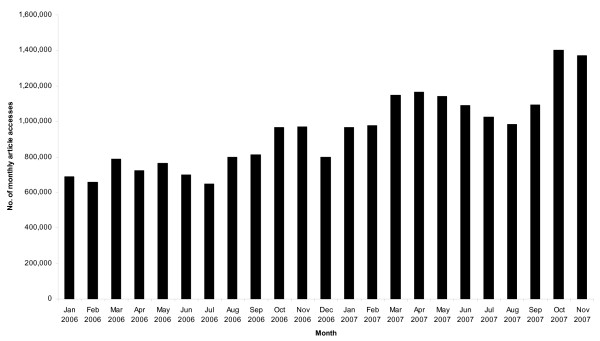
Number of monthly accesses of articles published in BioMed Central independent journals over time.

As a BioMed Central independent, open access journal, *Osteopathic Medicine and Primary Care *is uniquely positioned to serve its readers. In just the past two years, viewing of BioMed Central independent journals has doubled (Figure 1). This demonstrates that the extraordinary potential of open access publishing in attracting new readers and disseminating research findings is being realized. Because articles in *Osteopathic Medicine and Primary Care *are universally accessible online without charge, even clinicians and researchers in remote settings and those not affiliated with major universities may avail themselves of our latest publications. Thus far, 14% of all articles in *Osteopathic Medicine and Primary Care *have earned the "highly accessed" designation independently conferred by BioMed Central [2,3]. A second advantage of our open access publishing is the short lag time in bringing articles to press. During 2007, the mean time from initial manuscript submission to publication of peer-reviewed articles was 199 days. However, even this parameter is biased upwards because several manuscripts were already accepted but awaiting the official launch of *Osteopathic Medicine and Primary Care *in January 2007. When considering only those manuscripts initially submitted following our launch, the mean lag time to publication of peer-reviewed articles was 137 days. Our goal in 2008 will be to further reduce this lag time to less than 120 days. A third advantage of our open access publishing is that all articles are immediately indexed in PubMed and permanently archived in PubMed Central, the full-text repository of life science literature maintained by the United States National Library of Medicine. During 2007, *Osteopathic Medicine and Primary Care *was also accepted for indexing in CINAHL, the database providing a cumulative index to the nursing and allied health literature. Lastly, our open access publishing also provides unlimited space for appending tables, extensive data, figures, photographs, and video footage to an article. Nowhere in *Osteopathic Medicine and Primary Care *were such features more clearly evident in 2007 than in the highly accessed review by Hruby and Hoffman [2], which included 33 schematics and photographs illustrating various osteopathic manipulative treatment techniques in a manner reminiscent of an osteopathic textbook.

## Peer review and reader comments

*Osteopathic Medicine and Primary Care *strives to provide quick and fair review of all manuscripts. Our short lag time and quality of publications would not be possible without the voluntary efforts of dedicated reviewers. We are indebted to the 38 reviewers who have completed evaluations of submitted manuscripts to date (Appendix). While these reviews are crucial in selecting and improving the articles published in *Osteopathic Medicine and Primary Care*, we would like to know what readers think about our articles. The letter to the editor is the traditional mechanism for providing reader comments, including critical evaluations, of journal articles. Typically, however, few letters are published in response to any given article. Those highly selected letters that are published usually appear several months later than, and detached from, the relevant article. *Osteopathic Medicine and Primary Care *provides an opportunity for immediate reader comments on its articles. Readers may submit their comments to an article using either of the hyperlinks provided on the title page and last page in the full text version of the article (reader comment hyperlinks are not available in the PDF version of an article). Reader comments undergo editorial review to ensure appropriateness of content; however, our intent is to be broadly inclusive of reader comments rather than highly selective. Comments relating to clinical practice generally will not be posted without supporting evidence from peer-reviewed publications. Figures, tables, and equations cannot be included within a posted comment; however, hyperlinks to such elements may be included. Upon acceptance all reader comments are directly linked to the full text version of the relevant article. Reader comments are not indexed in PubMed or other bibliographic databases.

## Article processing charges and waivers

In the absence of revenues from subscriptions and commercial advertising, *Osteopathic Medicine and Primary Care *levies article processing charges (APCs) to cover the cost of operations. Authors at institutions that are members of BioMed Central will receive either a full or partial discount of the APCs depending on their institutional membership. During 2007, *Osteopathic Medicine and Primary Care *established a journal fund to provide waivers of APCs for authors who are not affiliated with BioMed Central member institutions and who do not otherwise have access to grant funds or local monies to support the cost of publication. The journal fund will consider APC waivers for eligible authors on a "first-come, first-served" basis. Once author eligibility is established, the two primary criteria in determining whether a waiver is awarded are the quality of the submitted manuscript and its congruence with the journal's scope of publication.

## Conclusion

*Osteopathic Medicine and Primary Care *affords authors the opportunity for rapid and universal dissemination of their work. We are keen to receive author manuscripts and reader comments on articles during 2008. A journal fund has been established to offset the cost of APCs for eligible authors who submit qualified manuscripts.

## Competing interests

The author(s) declare that they have no competing interests.

## Appendix

*Osteopathic Medicine and Primary Care *peer reviewers through 2007: Bruce Bates, Francis Blais, Pablo Calzada, Roberto Cardarelli, Mark DeHaven, Robert DiTomasso, Charlotte Foley, Thomas Flood, Kimberly Fulda, John Gimpel, Stanley Grogg, Peter Gulick, John Howell, Robert Jones, Hollis King, Michael Kuchera, Shrawan Kumar, Grace Kuo, Margot Kurtz, Emil Lesho, John Licciardone, Sue Lurie, Robert Moran, Reza Nassiri, Kenneth Nelson, Donald Noll, Ed Owens, Matthew Ridd, Paula Scariati, Michael Seffinger, Samuel Snyder, Karen Steele, Carolyn Tarrant, Kevin Treffer, Shwu-Fen Wang, Richard Virgilio, Robert Woodworth, David Yens.
